# Novel Benchtop Magnetic Particle Spectrometer for Process Monitoring of Magnetic Nanoparticle Synthesis

**DOI:** 10.3390/nano10112277

**Published:** 2020-11-17

**Authors:** Norbert Löwa, Dirk Gutkelch, Ernst-Albrecht Welge, Roland Welz, Florian Meier, Abdulkader Baki, Regina Bleul, Thorsten Klein, Frank Wiekhorst

**Affiliations:** 1 Physikalisch-Technische Bundesanstalt, Abbestr. 2-12, 10587 Berlin, Germany; Dirk.Gutkelch@ptb.de (D.G.); Ernst-Albrecht.Welge@ptb.de (E.-A.W.); Frank.Wiekhorst@ptb.de (F.W.); 2 Postnova Analytics GmbH, Max-Planck-Straße 14, 86899 Landsberg am Lech, Germany; Roland.Welz@postnova.com (R.W.); Florian.Meier@postnova.com (F.M.); Thorsten.Klein@postnova.com (T.K.); 3 Fraunhofer Institut für Mikrotechnik und Mikrosysteme IMM, Carl-Zeiss-Straße 18-20, 55129 Mainz, Germany; Abdulkader.Baki@imm-extern.fraunhofer.de (A.B.); Regina.Bleul@imm.fraunhofer.de (R.B.)

**Keywords:** magnetic nanoparticles, synthesis, magnetic particle spectroscopy, magnetic characterization, process monitoring, biomedical applications

## Abstract

Magnetic nanoparticles combine unique magnetic properties that can be used in a variety of biomedical applications for therapy and diagnostics. These applications place high demands on the magnetic properties of nanoparticles. Thus, research, development, and quality assurance of magnetic nanoparticles requires powerful analytical methods that are capable of detecting relevant structural and, above all, magnetic parameters. By directly coupling nanoparticle synthesis with magnetic detectors, relevant nanoparticle properties can be obtained and evaluated, and adjustments can be made to the manufacturing process in real time. This work presents a sensitive and fast magnetic detector for online characterization of magnetic nanoparticles during their continuous micromixer synthesis. The detector is based on the measurement of the nonlinear dynamic magnetic response of magnetic nanoparticles exposed to an oscillating excitation at a frequency of 25 kHz, a technique also known as magnetic particle spectroscopy. Our results underline the excellent suitability of the developed magnetic online detection for coupling with magnetic nanoparticle synthesis based on the micromixer approach. The proven practicability and reliability of the detector for process monitoring forms the basis for further application fields, e.g., as a monitoring tool for chromatographic separation processes.

## 1. Introduction

The unique magnetic properties of magnetic nanoparticles (MNP), combined with the ability to modify their surface chemistry, make them ideally capable for a variety of biomedical applications [1]. Therapeutically, MNP are applied in hyperthermia and targeted drug delivery, while for diagnostic purposes they are employed as contrast agents in magnetic resonance imaging (MRI) and magnetic particle imaging (MPI). The latter is a novel quantitative imaging technology with potential for cancer diagnosis using MNP as local probes [2].

The magnetic properties of the MNP and, consequently, their intended behavior in the envisaged application depend on a complex interrelationship between size, morphology, structure, and surface modification of the fabricated particles [3,4,5,6]. Therefore, magnetic measurement techniques are indispensable for quality assurance, research, and development of MNP.

So far, conventional batch methods such as micro emulsion, hydrothermal reactions, sol-gel, and coprecipitation have been commonly used to synthesize iron oxide MNP. However, reaction control is generally limited, and multistep synthesis routes require post treatment such as washing and transfer from organic to aqueous phases. Therefore, these approaches suffer from high batch-to-batch variation and low reproducibility of particle characteristics.

To surmount these obstacles, the microtechnological manufacturing approach for the controlled synthesis of magnetic iron oxide nanoparticles via aqueous synthesis route was developed (micromixer synthesis) [7]. One main advantage of the micromixer synthesis platform is the spatial and temporal separation of nanoparticle nucleation and growth. The main component of the set-up is a micromixer device where a homogenous nucleation of MNP is induced, to which locally separated dwell zones, often called residence loop, for an optimal control of particle growth are connected [8]. Core seeds formed at first contact of the educts in the micromixer are then piped into the residence loop where subsequent particle ripening takes place, e.g., the cores grow to a certain size depending on the time as well as the temperature in the residence loop.

Adjusting individual process control parameters, e.g., synthesis temperature and residence time, allow for accurate process control of the micromixer setup. A detailed description of micromixer synthesis for reproducible production of single-core iron oxide MNP with defined magnetic properties is given in an article by Baki et al., in the same journal [9]. To fully exploit the potential of the micromixer synthesis, a comprehensive understanding of the process parameters and their influence on the magnetism of MNP is essential. The availability of appropriate measurement technology for real-time monitoring of the magnetic functionality of MNP during synthesis would significantly accelerate MNP development and at the same time would provide the necessary technology for automatic process control and quality assurance.

Although several methods for monitoring the synthesis of nanoparticles have already been developed and applied [10,11], there is no detector available capable to monitor the essential magnetic functionality of MNP in line with a micromixer setup. The basic utility of inline monitoring of micromixer MNP synthesis has been demonstrated by Bemetz et al., who used a nuclear magnetic resonance (NMR) device to determine the transversal and longitudinal relaxation times of MNP [11]. Although NMR is only an indirect method for evaluating the magnetic properties of MNP, valuable information for synthesis optimization of MRI contrast agents was obtained. Furthermore, the high static magnetic field of 0.5 T required for the detection might raise the danger of aggregation or chain formation of the MNP during the NMR measurements.

Recently, a magnetic detector for batch synthesis monitoring in a 100 mL reacting flask has been presented [12]. Although this system is not designed for flow measurement, it operates at an excitation field strength of a few millitesla, making aggregation very unlikely. The method used is called magnetic particle spectroscopy (MPS), which is a robust and fast technique for the sensitive detection of MNP using conventional coils. It is based on the detection of the nonlinear dynamic magnetic response of the MNP exposed to a sinusoidal excitation field at a fixed frequency in the lower kilohertz range and an amplitude of tens of a millitesla. Originally developed for assessing the MNP performance in MPI, the MPS technique rapidly showed enormous potential in characterizing and quantifying the MNP content in tissue, blood, or cells [13,14,15,16]. Usually, MPS uses a sinusoidal magnetic field of fixed excitation frequency and sufficiently high amplitude to drive the MNP magnetization towards saturation. Consequently, the Fourier transform of the measured magnetization response contains odd multiples of the excitation frequency. Since the MNP response is a consequence of an alternating magnetic field, the dynamics of the magnetization reversal plays a decisive role in MPS. The magnetization reversal of MNP is governed by two distinct relaxation effects, the Néel relaxation caused by rotation of magnetic moments within the core crystal and the Brownian relaxation by rotation of the entire particle. Therefore, the relaxation processes are directly linked to structural properties (e.g., anisotropy, crystal structure) and physical conditions (e.g., viscosity of the medium, binding state) of the MNP, which forms the basis for a number of MNP applications. For example, magnetic hyperthermia therapy is based on local heating of the MNP environment caused by the magnetic moment, which lags behind the excitation by the Néel processes and shows hysteresis losses [17]. In bioassays using magnetic markers, Brownian relaxation differs from analytically bound and unbound markers, which can also be detected using MPS [18]. In contrast, MPI uses nanoparticles that are either in the Néel regime, where the signal is a function of the number of MNPs, only, or in the Brownian regime, where local viscosity and binding to the environment shall be visualized [19].

Therefore, three characteristic parameters of the MPS harmonic spectra, the amplitude of the third harmonic normalized to the iron amount of the sample, *A*_3_*, the (concentration independent) ratio between 5th and 3rd harmonic, *A*_5_/*A*_3_., and the phase of the 3rd harmonic *φ*_3_ are considered. All values are correlated to the MPI performance with the general observation that the higher the *A*_3_*, and *A*_5_/*A*_3_, the better the MPI images. The phase *φ*_3_ describes how well the particle moments can follow the excitation field at the given frequency *f*_0_. Generally, it is observed that the smaller the particle, the better the magnetic moments follow the excitation field and the closer the value of *φ*_3_ is to zero.

Here, we present the capability and performance of a sophisticated benchtop MPS device to magnetically monitor the continuous, targeted production of MNP by micromixer synthesis. One main challenge of such a magnetic detector is to provide a high sensitivity (e.g., a low detection limit), large dynamic range, and high temporal resolution in determining magnetic parameters of MNP. This enables us to control the synthesis process of MNP over an as-large-as-possible range of synthesis parameters. On the other hand, depending on the field of intended application of the MNP, it might be advantageous to record the full dynamic magnetization response of the particles, e.g., to include the signal measured at the excitation frequency at a price of reduced dynamic range. Therefore, the benchtop MPS comes along with two operation modes, which are mainly selected by a band stop filter to suppress excitation signals in the detector. In addition, the benchtop MPS device comprises a simple and regularly repeatable calibration procedure. The measured MPS signals of MNP obtained during the micromixer synthesis are compared with measurements of Ferucarbotran made with our benchtop MPS device. Ferucarbotran, which is a precursor of the liver MRI contrast agent Resovist^®^, is considered as a gold standard with high and comparatively stable MPS performance.

## 2. Materials and Methods

### 2.1. MPS Device

**Figure 1:**
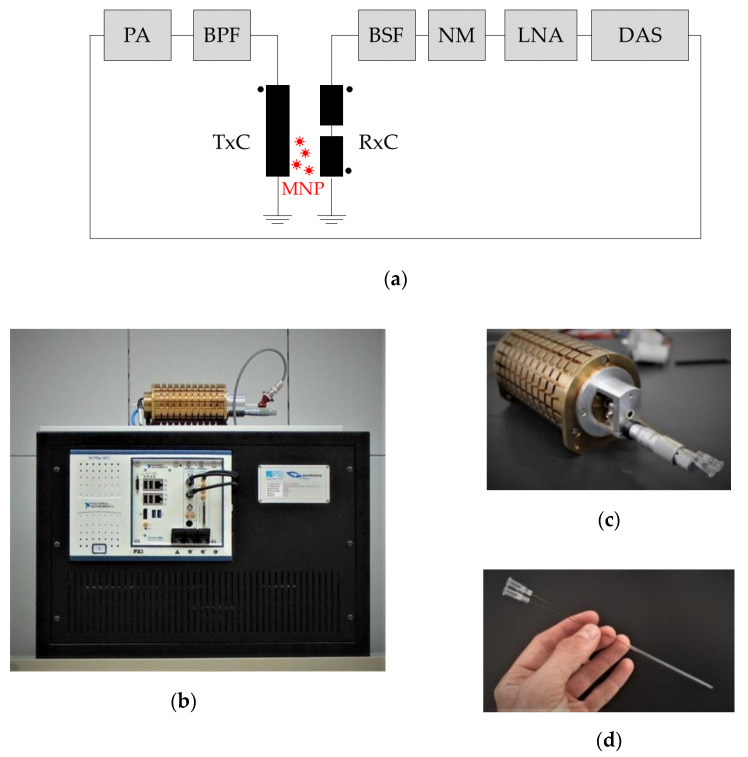
Benchtop magnetic particle spectroscopy (MPS) device: (**a**) Schematic signal chain of the benchtop MPS device consisting of a power amplifier (PA), a band-pass filter (BPF), and a transmit coil (TxC) on the excitation path. The receive path consists of a connectible band-stop filter (BSF), a noise matching transformer (NM), a low noise amplifier (LNA), and the data acquisition system (DAS). (**b**) Complete MPS device with computer-controlled data acquisition system (white chassis) and coil housing (top) without protective shielding. (**c**) Receive and transmit coil housing with inserted flow cell. (**d**) Capillary flow cell with theta-cross-section and polyimide-reinforced glass capillary inlet and outlet equipped with Luer-Lock connections.

Generally, an MPS system is composed of a transmit unit to produce the sinusoidal magnetic excitation field; a receive unit to filter, detect, and amplify the MNP response; and a control unit with data acquisition and analysis—a schematics of the MPS device components is shown in Figure 1a. Our benchtop MPS device (a photograph is shown in Figure 1b) consists of a transmit (TxC) and a receive coil (RxC) unit connected to a computer-controlled data acquisition system. Both coil systems are encapsulated by a brass cylinder (Figure 1c) used for both the electrical shielding of electromagnetic interferences and cooling. The data acquisition system comprises an embedded controller system (National Instruments, PXIe-8880, Munich, Germany), an arbitrary waveform generator (AWG, National Instruments, PXI-5421, Munich, Germany), and a 24-bit digitizer board (National Instruments, PXI-5922, Munich, Germany).

#### 2.1.1. Excitation Path

Litz wire (Rupalit V155, 2 × 521,500 × 0.05, Pack Feindrähte GmbH, Gummersbach, Germany) was used for the TxC (length: 100 mm, inner diameter: 9 mm, 4 layers, 132 overall turns) to provide a homogeneous magnetic field (deviation max. 0.7%) over 20 mm of the inserted flow cell at amplitudes up to 50 mT. The transmit coil is air-cooled by means of a diaphragm vacuum pump (Air Jet, Bürkle, Bad Bellingen, Germany) to maintain stable temperature conditions (310 K) within 30 min with an excitation field amplitude *B* of 12 mT permanently switched on. A calibrated single turn coil surrounding the transmit coil is used to control the excitation field amplitude. Additionally, the monitoring coil signal serves as a reference for the receive coil phase signal. A third order passive band-pass filter with a center frequency of 25 kHz and a bandwidth of 10 kHz provides more than 50 dB attenuation of frequencies below and above the center frequency (e.g., 75 kHz, third harmonic). To maximize the power transfer, a capacitive voltage divider is used to match the impedance of the TxC to the output impedance of the power amplifier used (Dynacord SL 2400, Thomann GmbH, Berlin, Germany. The input of the power amplifier is fed with a sinusoidal signal from the AWG (see Section 2.1.3).

#### 2.1.2. Signal Acquisition and Excitation Cancellation

**Figure 2:**
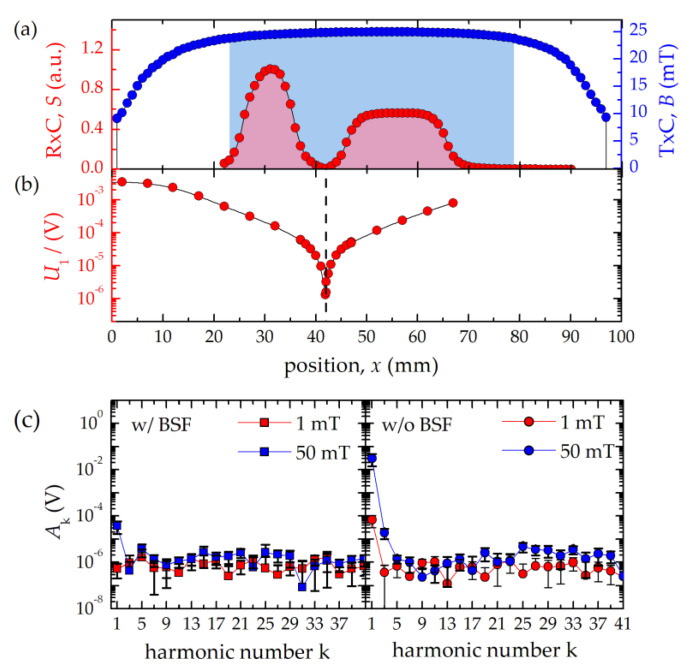
Signal acquisition and excitation cancellation with benchtop MPS device: (**a**) Axial excitation field profile (blue symbols) inside the TxC measured by a small probing coil (inner diameter: 1.5 mm, length: 1.7 mm, four layers, 68 turns). The range in which the excitation field deviates from the maximum value by less than 5% is highlighted in blue. The probing coil was further used to generate a reference signal to determine the sensitivity profile of the receive coil (red). (**b**) Measured induced voltage (1st harmonic) at different positions of the RxC within the TxC. (**c**) Measured spectrum of an empty flow cell (averaging 0.2 s, with background correction) at 1 mT (red symbols) and 50 mT (blue symbols) excitation field strength with (left, squares) and without (right, circles) BSF. Note, the connecting lines are a guide to the eye.

The gradiometric RxC (inner diameter 4 mm) consists of a receiving part (coil length 20 mm, one layer, 72 turns) and an oppositely wound compensation part (coil length 10 mm, two layers, 72 turns) to ensure suppression of the strong excitation signal at 25 kHz (Figure 2a–c). For this purpose, the tip of the flow cell is positioned into the receiving part, only while the compensation coil remains empty. Nevertheless, the remaining cross coupling signal of the mutually coupled coils still limits the dynamic range of the detector. To further reduce the excitation feed-through at *f*_0_, a passive band-stop filter (BSF) has been developed, which provides an attenuation of 60 dB (Figure 2c) extending the maximum dynamic range spanning over 6 orders of magnitude. The use of the BSF is optional, thus, without BSF, the detection of MNP signals including the first harmonic is possible, e.g., for the reconstruction of the time-dependent magnetization curve. The remaining voltage from the sensing system is amplified by 40 dB through a differential low-noise pre-amplifier (LNA, 2/4/6-C, Physical Acoustics Corp., Princeton Junction, NJ, USA) with 15 pF input capacitance and an integrated band-pass filter (bandwidth 30 kHz–700 kHz). As the LNA is still noise dominant (2.4 nV/√Hz) compared to the 2.4 Ω RxC with a noise level of 203 pV/√Hz, a custom-built noise matching transformer with a turn ratio of six is connected to the LNA (Figure 1a) which amplifies the signal by 15.6 dB [20,21].

#### 2.1.3. Signal Generation and Data Acquisition

For the excitation signal generation, a digital sine signal consisting of 16,000 points is generated in the memory of the AWG. As the sample rate of the AWG is *fs* = 100 MHz, four periods are stored to get an output frequency of *f*_0_ = 25 kHz (i.e., 4000 points per period). The measured signal from the receive and the monitoring coil path is recorded with the two-channel digitizer at a sampling frequency of *fs* = 5 MHz. Data are read in blocks of 100,000 values (32-bit signed integer) resulting in a minimum measuring time of t = 20 ms for each path and a memory requirement of 4 MB per channel. For the calculation of the time average, blocks of 100,000 points are averaged over sections of 2000 points (correspond to 10 periods of *f*_0_), which results in averaging over 50 values each. The time average is processed as double value (8 byte) during the running measurement resulting in a memory requirement of 16 kB per channel. The reference clock of the digitizer is the PXI system reference clock to ensure time synchronicity of AWG and digitizer. This achieves the phase-locked coupling of the AWG and the digitizer. Subsequently, a discrete Fourier transform (DFT) is applied to both channels resulting in a frequency resolution of 2.5 kHz. The information-carrying harmonics appear as multiples of 10 bin in the resulting DFT spectrum. The phase is referred to the zero phase of the fundamental wave recorded by the monitoring coil. Signal distortions caused by the RxC, (optional) band-stop filter, and pre-amplifier are compensated by multiplying the measurement data with a separately determined transfer function (see Section 3.1). Residual background signals of a recorded empty MPS measurement are subtracted from all datasets. The frequency-dependent uncertainty of the harmonic amplitudes is determined by 20 repeated measurements of an empty MPS. The standard deviation was calculated to quantify the uncertainty of the measurement process using a coverage factor of k_c_ = 1. The limit of detection (LOD) is determined according to guidance of the International Union of Pure and Applied Chemistry (IUPAC) as mean + 3 times the standard deviation of empty sample holder measurements with a coverage factor of k_c_ = 1, as well.

To analyze the MNP magnetization dynamics and saturation effects, a reconstruction of the dynamic magnetization curve and the point spread function is performed according to [22].

#### 2.1.4. Flow Cell

A purpose-built flow cell (outer diameter 2.9 mm, effective inner diameter 2.08 mm) consisting of a theta-cross-section capillary (Hilgenberg GmbH, Malsfeld, Germany) with an end cap for reversing the flow direction was used to continuously measure MNP in liquid flow (Figure 1d). The inlet and outlet consist of polyimide-reinforced glass capillaries (outer diameter 0.5 mm), which are equipped with Luer-Lock connectors. The theta-cross section capillary of the flow cell is positioned inside the detecting RxC with an active volume of 53.8 µL. An influence of the flow velocity on the measurement is not to be expected with a given measuring frequency of 25 kHz, since the area of homogeneous field of the TxC is sufficiently larger than the sensitive area of the RxC. For example, with the given flow cell geometry and a flow rate of 10 mL/min, the particles would travel a distance of 4 µm after a period of excitation (40 µs). Before entering the sensitive area of the RxC (see Figure 2a), the particles have already been in an excitation field of constant amplitude for about 2550 periods (i.e., a distance of 10 mm). In addition, the influence of the flow rate on the magnetization dynamics of MNP is below 5% [23].

### 2.2. Chemicals and Samples for Calibration

We used two commercial MNP systems, Ferucarbotran (FER, Meito Sanyo Co., Nagoya, Japan), and EFH-3 Ferrofluid (EFH, FerroTec, Santa Clara, CA, USA).

FER is the precursor of the liver MRI contrast agent Resovist^®^ and appreciated as a gold standard due to good dynamic magnetic properties in MPS. Though Resovist has been withdrawn from the market, the precursor Ferucarbotran offering the same magnetic properties can be purchased from Meito Sanyo, Japan. FER consists of an aqueous suspension of single and multi-core iron oxide nanoparticles coated with carboxydextran. A dilution series of FER in demineralized water with the resulting concentration of 33.5 mg/mL, 522 µg/mL, 52.2 µg/mL, and 3.7 µg/mL was prepared and filled into the MPS flow cell.

EFH is a light hydrocarbon oil-based ferrofluid developed for technical applications (audio speaker, dampers, sealings). We used 3D printing by the digital light processing technique to produce long-term stable cylinders (diameter 2.9 mm, height 8 mm) containing a fraction of EFH nanoparticles. Using ultrasonication, the EFH particles were homogeneously embedded into a photopolymer resin (R05 red, Envisiontec GmbH, Gladbeck, Germany) to a final iron concentration of *c*(Fe) = 4.5 mg/mL, which was then cured by light during printing.

For calibration and for assessment of the performance of our benchtop MPS system, we used a commercial MPS system (MPS-3, Bruker BioSpin, Ettlingen, GER) operating at *f*_0_ = 25 kHz and *B* = 10 mT amplitude. This system outputs the harmonics in units of magnetic moment (Am^2^) calibrated by a traceable coil (VSL, Dutch National Metrology Institute) provided with the MPS system.

### 2.3. Micromixer Synthesis

Single-core iron oxide nanoparticles were synthesized in a micromixer setup by precipitation from aqueous solutions of divalent iron chloride, sodium nitrate as oxidizing agent, and sodium hydroxide (all reagents were used without further purification, purity grade ≥ 98%, Sigma Aldrich, Taufkirchen, Germany). Alkaline solution of di-valent iron salt [8], solutions of iron chloride, sodium nitrate as oxidizing agent, and sodium hydroxide (all reagents were used without further purification, purity grade ≥ 98%, Sigma Aldrich, Taufkirchen, Germany). The microfluidic synthesis platform consists of HPLC pumps (Knauer, Berlin, Germany), caterpillar micromixer (Fraunhofer IMM, Mainz, Germany) to induce particle nucleation and several temperature-controlled reaction loops to control particle growth and was enhanced by a downstream processing module to remove reactive agents and excess of stabilizing agent. Solutions of iron chloride, sodium nitrate as oxidizing agent, and sodium hydroxide were mixed in a caterpillar micromixer with symmetric liquid ratios and piped in a temperature-controlled reaction loop. In two consecutive synthesis runs, we either varied the synthesis temperature in the micromixer or the mixing ratio of the educts to demonstrate the impact of process parameters on the magnetic behavior of the resulting MNP as detected by our benchtop MPS. Therefore, the device was connected downstream to the micromixer platform using Luer-Lock connectors (Figure 1a). Details of the micromixer synthesis platform and the influence of synthesis parameters on the magnetic behavior of the MNP obtained by a thoroughly structural and magnetic characterization are found in [9] in the same journal.

## 3. Results and Discussion

### 3.1. Calibration of the Benchtop MPS System by Commercial MNP Systems

To provide an easy and regularly repeatable calibration procedure of the benchtop MPS, we tested the capability of the two MNP systems FER and EFH. First, a sample of 0.2 mg (Fe) liquid FER and a sample of 0.17 mg (Fe) 3D printed EFH were measured at 10 mT with the calibrated MPS device. Subsequently, the same samples were measured at the same excitation amplitude of 10 mT with the uncalibrated benchtop MPS device. For each sample, the transfer function, i.e., the ratio of the complex MPS spectra between the calibrated (in units Am^2^) and uncalibrated (in units V) device, was determined.

**Figure 3:**
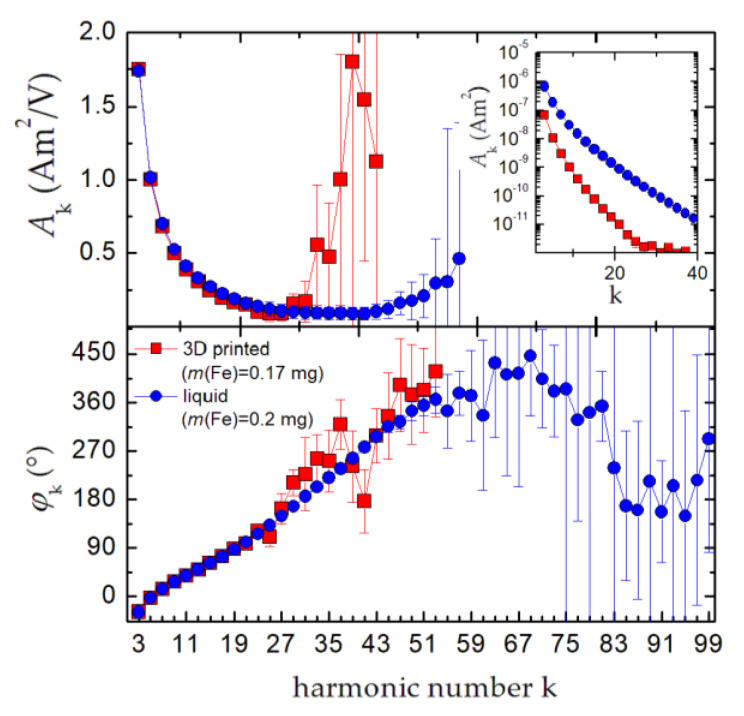
Transfer function of the benchtop MPS detector as determined by two different magnetic nanoparticle (MNP) systems: A robust and long-term stable 3D printed cylinder of EFH (red squares) and liquid suspension of FER (blue circles). Note, the connecting lines are a guide to the eye.

As can be seen in the inset of Figure 3, the FER sample is better suited, since it generates signals over a wider range of harmonics k·*f*_0_. Though both calibration samples contain about the same amount of iron, FER allows for calibration up to harmonic k = 51. Due to the steeper decrease in the spectrum and the lower signal amplitude (see inset of Figure 3), the 3D-printed EFH sample only covers a smaller frequency range up to k = 29 for calibration. This significant reduction in MPS amplitudes (normalized to the iron amount) observed in the EFH cylinders is attributed to the reduced mobility of the MNP moments compared with EFH or FER in a fluid suspension. However, the solid-state EFH cylinder is very well suited for calibration, since it is robust, long-term stable, and easy to handle [24]. Furthermore, with the homogenization process to embed the EFH particles in the photo polymer and subsequent 3D printing to produce the EFH cylinders, a reproducibility better than 2% can be achieved. Presently, the 3D printing of rigid samples with a homogeneous EFH distribution at higher MNP concentration *c*(Fe) > 0.17 mg is not feasible. Once available, this type of embedded MNP would be ideal for the production of reference and calibration samples. For the following experiments, we performed calibration solely with FER. The transfer function was implemented into the control software of the benchtop MPS system to provide absolute moment values (in units of Am^2^) of the measured spectra for all measurements.

### 3.2. Determination of the Full Dynamic Measurement Range

**Figure 4:**
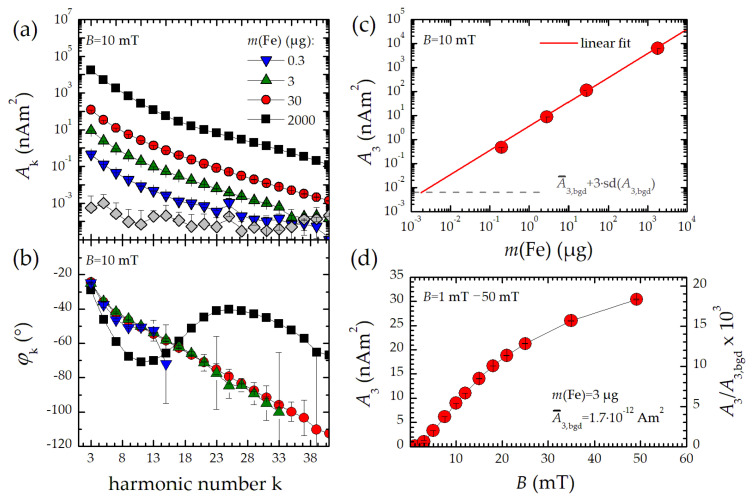
MPS signal of a serial dilution of FER: (**a**) MPS odd harmonic amplitudes; (**b**) odd harmonic phases; (**c**) concentration dependent 3rd harmonic amplitude *A*_3_ (uncertainties below 1%); (**d**) excitation field amplitude dependent third harmonic amplitude *A*_3_ of a *m*(Fe) = 3 µg FER sample (uncertainties below 1%). The right axis in the figure expresses the ratio between sample *A*_3_ and background signal *A*_3,bgd_ of the measured third harmonic. Note, the connecting lines in (**a**,**b**,**d**) are a guide to the eye.

To determine the dynamic range of the benchtop MPS device, a serial dilution of FER over a concentration range from *c*(Fe) = 34 mg/mL to 37 µg/mL was prepared and measured in the centered flow cell at *B* = 10 mT for 0.2 s. We used the BSF in the receive path to reduce the excitation feed-through at *f*_0_. Therefore, only odd harmonics *A*_k_ with k ≥ 3 were obtained, as displayed in Figure 4.

We observed a linear decrease in harmonic amplitudes with decreasing iron content without affecting the shape of the MPS spectrum and thus the dynamic magnetization behavior of the MNP (Figure 4a). Only for the highest concentrated sample (black squares) a slight change in the signal shape in the amplitude spectrum was observed. This change was even more apparent in the phase spectrum (Figure 4b), even though the digitizer input was not saturated. This might be attributed to dipole interactions between the MNP at high concentrations, which were already reported for FER in a previous work [25]. However, a linear decrease in the 3rd harmonic amplitude *A*_3_ with iron content was measured. With a detection limit of 6.6 × 10^−12^ Am^2^, this corresponds to a minimum detectable iron mass of 1.4 ng at *B* = 10 mT excitation field. This demonstrates the outstanding dynamic range of more than five orders of magnitude of the benchtop MPS system if applying the BSF. Without this filter, the dynamic range is reduced by two orders of magnitude, while now, the full dynamic information including *A*_1_ is accessed (see Section 3.3).

In addition, the excitation field amplitude *B* directly influences the nonlinearity of the dynamic MNP response and can be adjusted from zero to 50 mT in the benchtop MPS device. The behavior of *A*_3_ as a function of *B* for FER is displayed in Figure 4d. Obviously, the dynamic magnetization behavior of FER tends to saturate at higher fields.

### 3.3. Full Spectral Information Measurement

**Figure 5:**
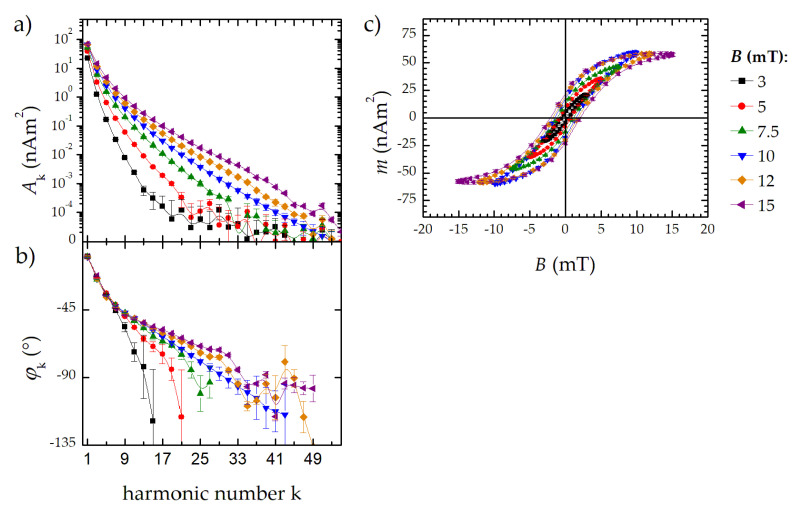
Field dependent MPS signal without *A*_1_ filtering: (**a**) MPS amplitude signal; (**b**) MPS phase signal; (**c**) reconstructed *M*(*H*) curve. The displayed lines are guides to the eye.

The measurement of the particle response at the excitation frequency *f*_0_ provides valuable information about the dynamic magnetization behavior of MNP. Additionally, to the “full dynamic range” mode (3.2), the benchtop MPS can be operated in a second mode without BSF, so that the first harmonic A_1_ is detected as well. Note that only MNP signals with amplitudes above the variability in the background and digitization error of the ADC can be detected (Figure 2c). This requires sufficiently high MNP concentrations or measuring at high excitation field amplitudes. To demonstrate the MPS performance in the “full spectral information” mode, we measured FER (iron mass *m*(Fe) = 2.8 µg) at different field amplitudes. No significant feed-through of the excitation was observed in the amplitude and phase signal at field amplitudes of *B* = 3 mT up to 15 mT (Figure 5a,b). Therefore, the dynamic magnetization curves *M*(*H*) can be reconstructed (Figure 5c). From the area of the *M*(*H*) curve (e.g., the hysteresis of the MNP), the heating efficiency of MNP in magnetic hyperthermia can be determined.

### 3.4. Synthesis Monitoring

To demonstrate the efficiency and performance capacity of the benchtop MPS in the micromixer approach, we performed two synthesis runs in which the changes in magnetic properties of the continuously produced MNP due to modifications of synthesis parameters are directly monitored by MPS. In a first synthesis run, the flow rate ratio of the educts were varied (see Section 3.4.1). Generally, the higher the total flow rate, the faster the nucleation of MNP seeds and, thus, the smaller the particles. However, changing the flow rate ratio of educts not only impacts the size but also the composition and quality of the resulting MNP. In the second synthesis run, the synthesis temperature is stepwise altered. For both experiments, we used a reduced excitation field amplitude of 12 mT of the benchtop MPS to avoid any heat transfer from the excitation coil of the online MPS device interfering the nanoparticle synthesis. Additional experiments are envisaged to further characterize if any heat transfer effects of the coil operation at higher field amplitude impacts the micromixer synthesis in the growth stage tubing.

#### 3.4.1. Changing the Flow Rate Ratio

**Figure 6:**
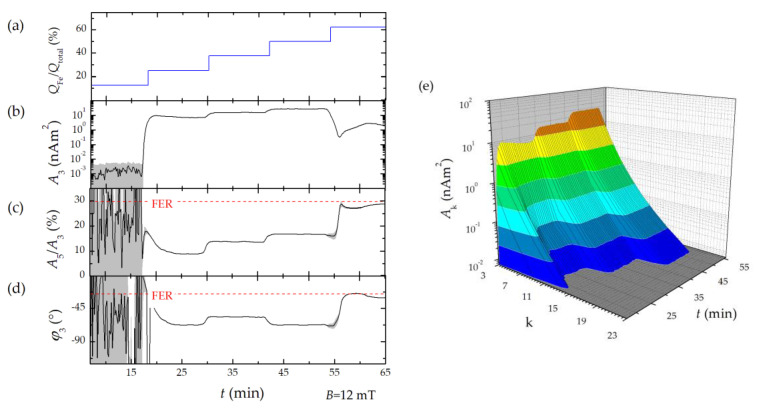
Inline benchtop MPS monitoring of micromixer MNP synthesis. (**a**) Time-dependent changing of iron to OH flow rate ratio. (**b**) Mean of MPS signal parameters *A*_3_, (**c**) *A*_5_/*A*_3_, (**d**) *φ*_3_ (black lines) and standard deviation (grey shaded area) of 10 consecutive measurements. The dotted red line highlights the values of FER. (**e**) Surface plot of time dependent MPS spectra.

To monitor the influence of varying the mixing ratio on the magnetic properties of the MNP directly during their micromixer synthesis, we operated the benchtop MPS with BSF, providing the largest dynamic range (see Section 3.2) but discarding *A*_1_. In the first experiment, the ratio of the flow rates of iron salt *Q*_Fe_ and alkaline *Q*_OH_ educt solutions was varied stepwise from *Q*_Fe_/*Q*_total_ = 12.5 to 62.5% to impact the MNP synthesis (Figure 6a). MPS measurements were performed at *B* = 12 mT with a temporal resolution of 1.2 s.While no MPS signals above the noise floor were detected for the lowest flow rate ratio of 12.5% (Figure 6b), a clear MPS signal of MNP was measured for all subsequent higher ratios (Figure 6b–d). Since *A*_3_ depends on both the MNP properties and the MNP amount in the detector coil, this value cannot be used to estimate the MNP concentration during the experiment. In contrast, *A*_5_/*A_3_* and *φ*_3_ are independent of the MNP amount and therefore ideally suited to differentiate MNP properties.

With an increasing flow rate ratio, a gradual flattening of the MPS spectra was observed (Figure 6e), as parameterized by the MPS parameter *A*_5_/*A*_3_, which increases from about 9 to 27%. The highest *A*_5_/*A*_3_ value was measured at a flow rate ratio of 62.5%. The phase shift *φ*_3_ was smallest under these synthesis conditions with −25°. Lower flow rate ratios below 62.5% had no significant influence on the phase, which was about −60°. Interestingly, at a flow rate ratio of 62.5% the measured MPS signals are very close to those of FER. This implies that the relative increase in iron salt fraction (compared to the alkaline educt solution) leads to synthesized MNP with a superior dynamic magnetism. Hence, the performance of the produced MNP promises a successful application in MPI as well.

#### 3.4.2. Varying the Synthesis Temperature

The synthesis temperature mainly determines the reaction velocity during the nanoparticle growth, since even at low temperatures, the nucleation is a very fast process. The influence of synthesis temperature variation has been investigated in detail in [9] for the micromixer setup used in this work. Here, we stepwise increased the synthesis temperature from 303 to 353 K and monitored the temperature impact on the magnetic behavior of MNP directly during the micromixer synthesis process. Therefore, we operated the benchtop MPS with the BSF (see Section 3.2) to obtain the largest dynamic range.

**Figure 7:**
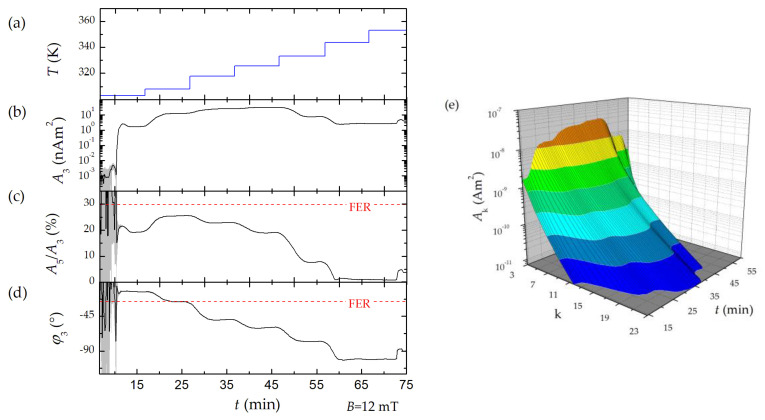
Inline benchtop MPS monitoring of micromixer MNP synthesis. (**a**) Time-dependent variation of synthesis temperature. (**b**) Mean MPS signal parameter *A*_3_, (**c**) *A*_5_/*A*_3_, (**d**) *φ*_3_ (black line) and standard deviation (grey shaded area) of 10 consecutive measurements. The dotted red line highlights the values of FER. (**e**) Surface plot of time dependent MPS spectra.

As displayed in Figure 7, different MPS signal changes were measured. In contrast to the previous experiment (Section 3.4.1), a monotonic decrease in *φ*_3_ with increasing synthesis temperature was detected. This might be due to an increase in the size of the MNP synthesized at higher temperatures [9]. As a consequence of the increasing MNP size, the resulting magnetic moments increase as well, which are then less able to follow the excitation field resulting in the observed increasing phase shift. In contrast, the *A*_5_/*A*_3_ ratio first increased for synthesis temperatures in the range from 303 K to 308 K, while for higher temperatures the *A*_5_/*A*_3_ ratio decreased again. Although a significant flattening of the spectra was observed (Figure 7e), the *A*_5_/*A*_3_ value of FER was not reached during the synthesis (Figure 7c). At a synthesis temperature of 308 K, the MPS signals were closest to those of FER. For this reason, a finer subdivision of synthesis temperatures in the range from 303 to 313 K would be useful to find out whether an increase beyond the performance of FER is possible. A flat harmonic spectrum (high *A*_5_/*A*_3_ value) and a low phase lag (i.e., low energy dissipation) are key parameters for a successful application in MPI.

The results clearly demonstrate that our novel benchtop MPS device is well suited to directly monitor the influence of the synthesis parameters and the magnetic quality of the produced MNP in the continuous micromixer synthesis. It should be noted that MPS primarily measures the dynamic magnetic properties of MNP during the synthesis. Hence, there is no direct access to geometric or structural parameters. Potentially, these parameters can be deduced from analysis of MPS data by either including models (e.g., to determine size distribution [26,27], viscosity [19,28]) or by combining with data obtained from other detectors operable during synthesis such as dynamic light scattering (DLS) or UV-Vis detector [29]. Though this generally should be feasible, the complexity of such an approach is rather high and might introduce large uncertainties. The situation could be improved by advancing the MPS technology to multi frequency and multi excitation field detection [26,30,31,32]. Therefore, we propose the use of MPS signals at this stage of development for continuous quality control during synthesis. In a next step of development, a correlation of MPS data (such as spectral shape parameter *A*_5_/*A*_3_ and phase *φ*_3_) with structural parameters should enable at least partial extraction of structural parameters from measured MPS data during synthesis. For this purpose, the benchtop MPS device must be operated in a controlled environment, as recently presented [29].

Besides mere magnetic analysis, it is advantageous to use additional detectors for the determination of structural information to obtain a comprehensive picture of the synthesized MNP and to derive detailed recommendations for MNP synthesis control. For example, additional structural information can help to determine which relaxation processes dominate the magnetic signal at a given excitation (Néel, Brown) and which MPI application fields (quantitative imaging, viscosity mapping) can be derived from it [19,33,34]. A suitable detector array could contain, e.g., small-angle X-ray scattering (crystallite size), DLS (hydrodynamic size), multiangle light scattering (core/cluster size), and/or UV-Vis (concentration). This approach has already been shown for the coupling with chromatography [23]. In this context, a multi-frequency acquisition scheme, as proposed in [30,35,36,37], would provide further valuable information about the frequency dependence of relaxation times and could be used for parameter estimation.

Nevertheless, MPS online analytics can already be used to adjust the magnetic properties of the MNP to further optimize MNP for specific biomedical applications. Each application requires MNP with specific magnetic (dynamic) properties. Used as a tracer for MPI, they should exhibit a dynamic magnetic behavior that shows MPS harmonic spectra with high amplitudes (*A*_3_ normalized to iron content) and a rich harmonic spectrum above the noise floor (large *A*_5_/*A*_3_ values). For heat production in magnetic hyperthermia application, MNP with large phase values *φ*_3_ are favorable.

## 4. Summary and Conclusions

We present a transportable benchtop MPS device designed for flow measurements and capable of measuring the dynamic magnetization behavior of MNP at a fixed excitation frequency of 25 kHz with high temporal resolution (20 ms). The device provides a large dynamic range spanning over six orders of magnitude. For FER, the detection limit was 1.4 ng, which is an improvement by factor three compared to our reference MPS system. If required, the device can also be used to measure the MNP signal at the excitation frequency in a second operating mode. In this case, however, a limitation of the dynamic range must be tolerated. We have also tested a simple calibration of the benchtop MPS device. For this purpose, we used a 3D printed magnetic reference body, which is suitable for calibrations up to the 29th harmonic. This enables the instrument to display harmonics in units of the magnetic moment (Am^2^).

We suppose that the benchtop MPS device could be well suited to monitor the magnetic properties of MNP during synthesis. Note, that for this first demonstration of the benchtop MPS performance, synthesis parameters have been chosen resulting in strong effects in the magnetic properties of the produced MNP. Sensitivity, temporal resolution, and online performance of the new device were evaluated. Conversely, relevant synthesis parameters can be identified directly during the continuous micromixer synthesis. Already based on the online measurements, we were able to derive recommendations for optimizing the MNP synthesis of suitable MPI tracers. Nevertheless, synthesis conditions cannot be derived from measured magnetic parameters at present. This requires further investigations such as those shown in [9]. In this way, micromixer platform and inline benchtop MPS constitute a powerful tool to propel future targeted MNP development. On the one hand, this enables the production of MNP for specific applications (e.g., MPI, hyperthermia) for which a specific signal behavior is desired (e.g., rich harmonic spectrum, dynamic hysteresis). On the other hand, different classes of MNP with defined signal properties can be synthesized on demand. This is advantageous, for example, for calibration and quality assurance tasks. In addition to synthesis monitoring, further fields of application open up, such as the monitoring of chromatographic separation processes [23] or magnetic blood purification [38], which are currently under investigation.

The outstanding sensitivity, large dynamic range, and short measurement time makes the online MPS technique a valuable tool for synthesis monitoring and could be used as a feedback loop to directly control the synthesis outcome in near future. This would require incorporating more features of the dynamic magnetic response to enable a unique correlation with synthesis parameters [9]. Presently, only the three main parametrizing values *A*_3_, *A*_5_/*A*_3_, and *φ*_3_ (the latter two with the advantage of being concentration independent) are considered in most investigations. Due to the extremely high sensitivity of the MPS technique, higher harmonics and harmonic ratios can be used to characterize and monitor magnetic parameters of MNP. Since MPS targets magnetic parameters, it could be used to reduce the influence of educt quality and starting material variations, improving the reproducibility and thereby safety of MNP in biomedical applications.
